# Oncocytic cell carcinoma of the thyroid with TERT promoter mutation presenting as asphyxia in an elderly: a case report

**DOI:** 10.3389/fendo.2024.1349114

**Published:** 2024-08-16

**Authors:** Xiqian Wang, Yingao Liu, Lijie Chen, Jie Zhang, Ruoyu Jiang, Lei Zhang, Han Yan, Jie Zhang

**Affiliations:** ^1^ Department of Nephrology, Tianjin Medical University General Hospital, Tianjin, China; ^2^ Department of General Surgery, Tianjin Medical University General Hospital, Tianjin, China; ^3^ Department of General Surgery, Jizhou District People’s Hospital, Tianjin, China; ^4^ Key Laboratory of Digital Technology in Medical Diagnostics of Zhejiang Province, Dian Diagnostics Group Co., Ltd., Hangzhou, Zhejiang, China

**Keywords:** thyroid neoplasms, carcinoma, asphyxia, mutation, aged

## Abstract

**Introduction:**

The prevalence of thyroid nodules and malignancies in the elderly is a growing concern. Thyroid nodules in this population have unique characteristics, requiring careful treatment strategies that balance risks and benefits. Oncocytic carcinoma of the thyroid (OCA) is a rare, aggressive subtype with diagnostic challenges.

**Methods:**

This case features an 84-year-old patient who presented with a neck mass and symptoms of asphyxia. Clinical evaluation, imaging studies, and biopsy were conducted to assess the nature of the thyroid lesion. Molecular testing, including genetic analysis, was performed to identify specific mutations associated with OCA and guide treatment decisions.

**Results:**

The patient was diagnosed with oncocytic carcinoma of the thyroid. The molecular testing revealed specific genetic mutations indicative of OCA, confirming the diagnosis. The presence of these mutations guided the treatment plan, emphasizing the importance of molecular diagnostics in managing thyroid malignancies, especially in the elderly.

**Discussion:**

This case illustrates the complexities of diagnosing and treating thyroid malignancies in the elderly. Biopsy and molecular testing provided diagnostic accuracy and informed treatment. Individualized approaches are essential for better outcomes, especially in aggressive subtypes, balancing the risks and benefits of intervention.

## 1 Introduction

As life expectancy increases, populations are rapidly aging, leading to a substantial rise in the elderly population. The prevalence of thyroid nodules increases with age, being detectable in 25–50% of individuals over 60 years old. However, the proportion of these nodules that are malignant in elderly individuals is comparable to, or even lower than, that in younger populations (1, 2). Compared to younger adults, elderly patients (≥65 years) with thyroid malignancies tend to have larger tumor volumes, higher proportions of Stage IV cases, and more extrathyroidal extension. Notably, the distribution of histological subtypes among elderly thyroid malignancies differs, with an elevated incidence of follicular thyroid carcinoma (FTC), OCA, medullary thyroid carcinoma (MTC), and anaplastic thyroid carcinoma (ATC) but a lower proportion of papillary thyroid carcinoma (PTC) (1, 2). Therefore, tumor progression is often faster in elderly patients, resulting in a poorer prognosis (3). Clinicians assessing thyroid nodules in elderly patients must carefully balance the potential harms of thyroid cancer overdiagnosis and the risks associated with delayed diagnosis to determine appropriate treatment strategies. In diagnosing and treating thyroid nodules in the elderly, comprehensive consideration of the patient’s circumstances is crucial. This includes a higher incidence of high-risk thyroid cancer, accompanied by multiple comorbidities, declining physiological function, cognitive abilities, and an increased risk of treatment complications. When making diagnostic and therapeutic decisions, a careful balance between the risks and benefits of thyroid nodule diagnosis and treatment must be exercised (4). This case report focuses on an elderly patient with oncocytic thyroid carcinoma presenting with asphyxia.

## 2 Case presentation

**Figure 1:**
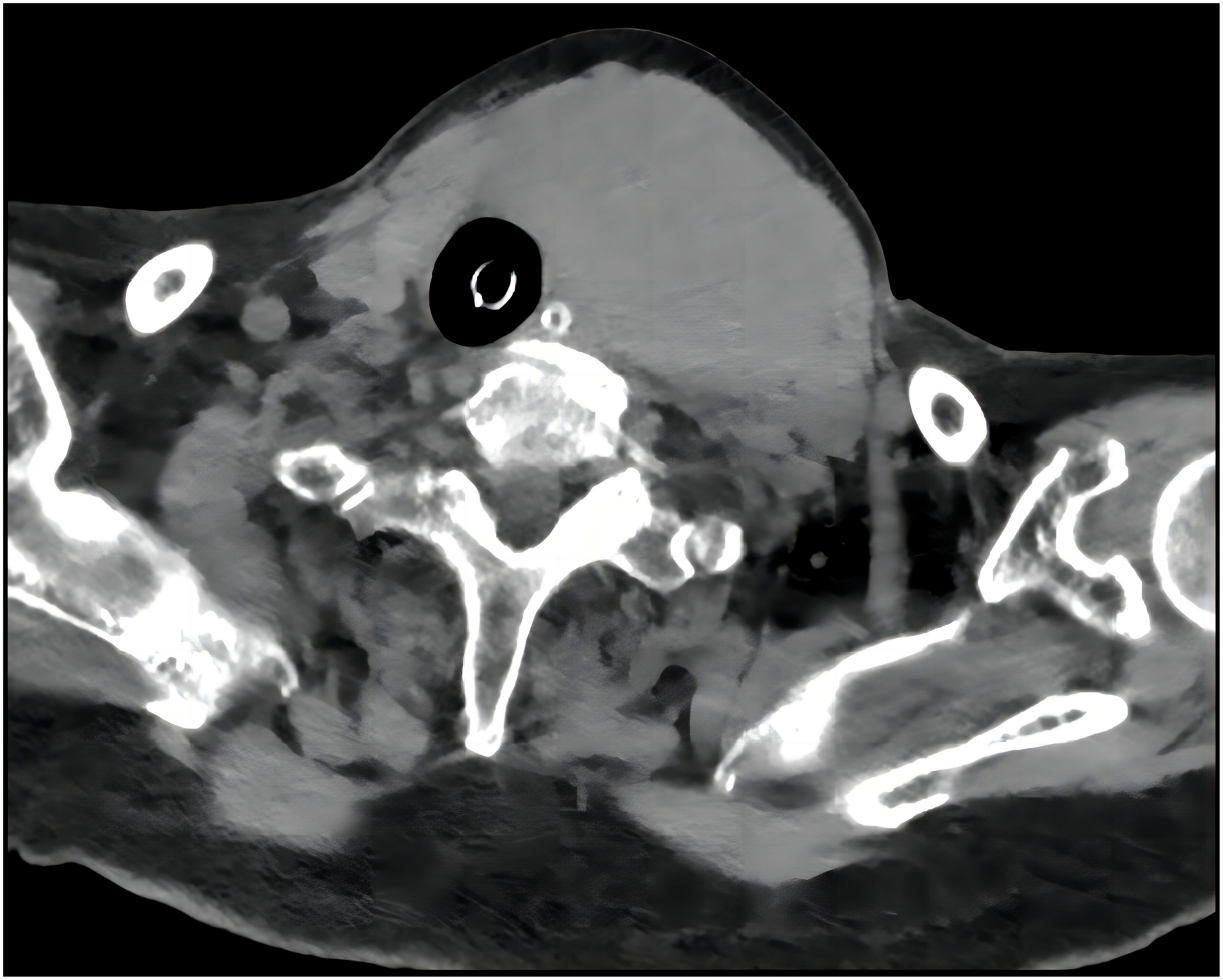
A CT of the neck revealed a solid lesion in the left thyroid lobe causing tracheal compression and deviation with endotracheal intubation.

**Figure 2:**
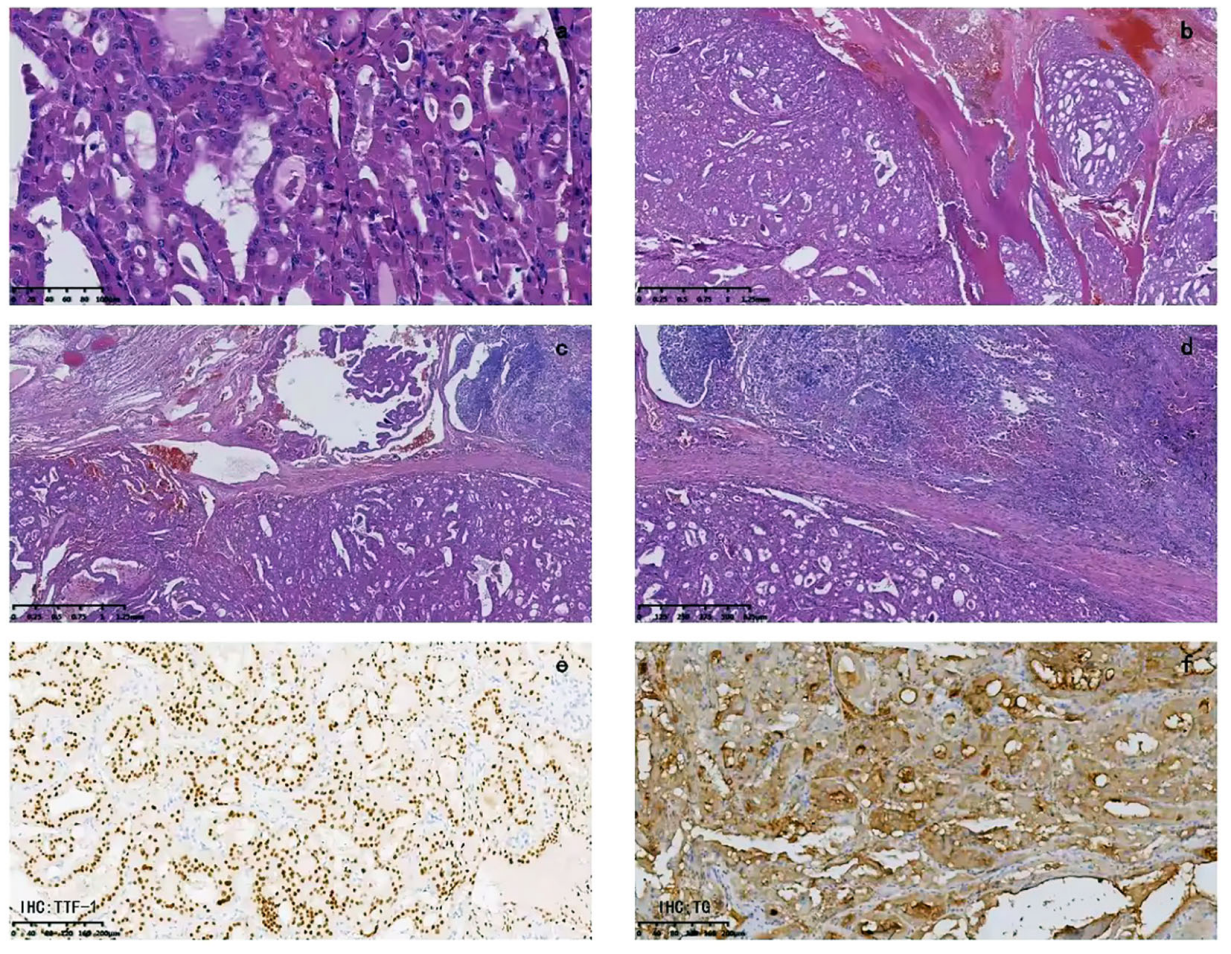
Pathological examination: **(A)** The tumor cells are cuboidal, mostly polygonal, with abundant cytoplasm filled with eosinophilic granular substances. The nuclei vary in size, being round, oval, or irregular in shape, with deep chromatin staining. Nuclear division can be seen, and nucleoli are visible in some areas. The cell boundaries are clear. The tumor cells are arranged in a follicular or trabecular pattern, with the follicular structure being predominant. There are areas with unevenly thickened colloid inside the follicular cavity. **(B)** Capsular invasion is visible, and outside the capsule, there are tumor cell nodules of varying sizes. **(C)** Tumor thrombi can be seen inside the vessels. **(D)** Focal necrosis is visible. **(E)** Immunohistochemistry for TTF-1 is positive. **(F)** Immunohistochemistry for TG is positive.

An 84-year-old female presented to the emergency department with asphyxia. Seven days before admission to our hospital, the patient with a history of hypertension, coronary heart disease, and paroxysmal atrial fibrillation for 3 years, developed a cough and productive sputum, along with dyspnea and choking sensation after drinking water. Her symptoms worsened, leading to respiratory distress and altered consciousness. The patient was initially treated at a local hospital with interventions including endotracheal intubation, mechanical ventilation, antimicrobial therapy, and nutritional support. Subsequently, the patient was transferred to our hospital and admitted to the Intensive Care Unit (ICU). Endotracheal intubation was maintained, connected to mechanical ventilation. Physical examination revealed clear consciousness, secured endotracheal intubation, a visible anterior neck mass with a soft consistency, fixed position, tracheal compression with right deviation, and no palpable enlargement of superficial lymph nodes in the anterior neck. Laboratory investigation showed thyroid stimulating hormone of 1.754 mIU/L (0.350-4.940), Free T3 of 3.40 pmol/L (2.43-6.01), and free T4 of 8.91 pmol/L (9.01-19.05). Neck ultrasonography indicated an enlarged left thyroid lobe with predominantly hyperechoic nodules containing vascularity, measuring approximately 7.2 × 3.8 × 6.9 cm (TI-RADS 3) in size. Contrast-enhanced computed tomography of the neck revealed a solid lesion in the left thyroid lobe causing tracheal compression and deviation, with endotracheal intubation (**Figure 1**). Because of the rapid progression, ultrasound-guided coarse needle biopsy (US-CNB) was performed to rule out ATC, revealing eosinophilic hyperplastic nodules. Molecular testing identified TERT promoter C228T mutations and negative results for BRAF, HRAS, KRAS, NRAS, TP53, RET, NTRK1, NTRK3, PAX8, and THADA. On the fourth day of admission, the patient underwent surgery under general anesthesia following the results of US-CNB. Intraoperatively, tumor compression of the left recurrent laryngeal nerve was detected, and due to the potential risk of bilateral recurrent laryngeal nerve injury leading to severe respiratory complications in consideration of the patient’s advanced age, only left thyroidectomy and isthmusectomy were performed, avoiding total thyroidectomy. The surgery was successful, and the endotracheal tube was removed on the second postoperative day. The patient had no respiratory distress but experienced hoarseness, likely due to tumor compression and intraoperative anatomical manipulation affecting the recurrent laryngeal nerve. The patient was discharged on the ninth postoperative day. Pathological examination of the resected specimen revealed an eosinophilic cell tumor of the thyroid with focal necrosis, suspected extrathyroidal extension, vascular tumor thrombus, indicating the possibility of oncocytic cell carcinoma. Immunohistochemistry demonstrated positive staining for CK19, TG, TTF-1, and CD56, negative staining for Galectin-3 and TPO, and positive CD34 vascular staining (**Figure 2**).

## 3 Discussion and conclusion

OCA, formerly known as Hürthle cell carcinoma (HCC), is a rare subtype of differentiated thyroid cancer and accounts for about 3-5% of all differentiated thyroid cancers (5–7). It is associated with poorer prognosis due to increased invasiveness and metastasis rates (6, 8). According to the WHO Classification for Thyroid Cancers 2022 (5th edition), the term “oncocytic carcinoma of the thyroid” is used to refer to invasive malignant follicular cell neoplasms composed of at least 75% oncocytic cells where the nuclear features of PTC and high-grade features are absent. OCAs are subclassified into minimally invasive (those with capsular invasion only), encapsulated angioinvasive, and widely invasive (those with gross invasion through the gland) tumors due to differences in clinical outcome (9). OCA typically occurs at an older age, with an average age exceeding 55 years, roughly 10 years later than the mean age of diagnosis for FTC. Although OCA is more common in women (with a 1.6 to 1 female-to-male ratio), it has a lower female-to-male ratio than is seen with FTC (6, 10). It usually presents as a slowly enlarging, painless, solitary thyroid nodule. Diagnosing OCA is challenging, as it can often be indistinguishable from benign oncocytic adenomas on thyroid ultrasonography. Differentiating between oncocytic adenomas and OCA through fine-needle aspiration biopsy (FNAB) is limited by sampling constraints (11).

Preoperative diagnosis of OCA presents significant challenges. A comprehensive diagnostic strategy can enhance accuracy, reduce missed and misdiagnosed cases, and provide better treatment options for patients. According to NCCN guidelines, preoperative evaluation should include neck ultrasound, CT, MRI, and molecular diagnostics to develop an optimal treatment plan. A retrospective study in South Korea on OCA revealed that among patients with confirmed OCA, 65.2% were classified as Bethesda Category IV, corresponding follicular neoplasm or suspicious for follicular neoplasm (FN/SFN), while atypia of undetermined significance or follicular lesion of undetermined significance (AUS/FLUS), suspicious for malignancy (SM), and malignant categories accounted for 8.7%, 5.4%, and 7.6%, respectively (12). Researchers have noted that managing thyroid nodules with indeterminate cytology, such as AUS/FLUS, remains a major challenge in endocrine pathology and thyroidology (13). The estimated risk of malignancy (ROM) for AUS/FLUS was 5-15% in the first edition of TBSRTC, which increased to 10-30% in the second edition, reflecting improved understanding and clinical management of these nodules (14). Recent studies suggest that the third edition of TBSRTC may further subdivide Category III into IIIA (without nuclear atypia) and IIIB (with nuclear atypia) to better reflect differing malignancy risks (14). This subdivision can help formulate more specific management recommendations based on the presence of nuclear atypia. Additionally, different management guidelines recommend fine-needle aspiration (FNA) for nodules of varying sizes. For instance, the 2015 American Thyroid Association (ATA) guidelines recommend FNA for nodules ≥10 mm with high-suspicion sonographic patterns to improve diagnostic performance and avoid unnecessary surgery (15). Larger cutoffs, such as 15 mm, may enhance the association between nodule size and malignant histopathology (15). In conclusion, subdividing cytological categories and incorporating molecular diagnostics play a crucial role in improving diagnostic accuracy (13–15).

OCAs have been shown to have recurrent DNA mutations, including RAS, EIF1AX, TERT, TP53, NF1, and CDKN1A genes (16–18). Research focused on follicular thyroid tumors indicated that TERT mutations are rare in follicular thyroid adenomas (FTA) but more common in FTC, and the presence of TERT mutations was associated with shorter patient survival, suggesting that TERT promoter mutations may serve as early genetic events occurring in follicular thyroid tumors, potentially leading to malignant transformation (19). TERT promoter mutations, specifically C228T and C250T, are mutually exclusive and more common in widely invasive OCAs than in minimally invasive cases (16, 20, 21).

Treatment decisions for elderly patients often prioritize conservative approaches due to shorter life expectancy, comorbidities, and higher surgical risks. However, differentiated thyroid cancers in the elderly tend to be more aggressive, with higher proportions of poorly differentiated or undifferentiated thyroid cancers, leading to adverse clinical outcomes. Thyroid cancer is the only one that takes into account the variable age in the staging of the disease, the AJCC Cancer Staging Manual (8th edition) designates an age of 55 or older as a high-risk prognostic factor. However, the lack of tools to predict high-risk thyroid malignancies in elderly individuals, especially those who are not suitable for biopsy, contributes to limited therapeutic strategies.

In the context of treatment, younger patients often pursue aggressive approaches to avoid future tumor progression or metastasis. Conversely, elderly patients, due to shorter life expectancy and comorbidities, face elevated surgical risks. Consequently, current guidelines advocate a conservative approach to assessing and treating thyroid nodules and thyroid cancer in elderly patients (22). Studies by Park also indicate a lower proportion of elderly patients opting for total thyroidectomy (23). However, among elderly individuals, differentiated thyroid cancer often exhibits greater invasiveness, and a higher proportion of poorly differentiated thyroid cancer is observed. Furthermore, elderly patients often demonstrate lesser concern about the development and progression of their tumors. Some patients may even present with clinical complications like airway obstruction or nerve damage due to tumor progression, leading to unfavorable clinical outcomes (24). Yet, assessing the prognostic risk of thyroid cancer in elderly patients remains limited, especially when diagnostic lobectomy is not suitable due to the patient’s condition, and methods for predicting high-risk tumors and applying such experience remain considerably constrained (25).

Our patient had discovered a neck mass two years before the presentation. Ultrasound showed an enlarged left thyroid lobe with a solid nodule measuring 58×40×31 mm (TIRADS-3). Although FNAB was indicated, the patient’s advanced age and associated risks led to the decision to forego further diagnostic procedures. Retrospective studies have shown that FNAB of thyroid nodules in patients aged ≥70 is safe and accurate for precise diagnosis (26). If FNAB had been conducted in this case, Bethesda Category III or IV, “follicular neoplasm” or “suspicious for follicular neoplasm,” might have been indicated. Even when US-CNB was performed, it was not possible to distinguish between benign and malignant based on the reason for sampling. Therefore, treatment decisions for surgery may still not be made. However, TERT promoter mutational detection through molecular testing could have influenced treatment decisions by identifying potential malignant or precancerous oncocytic tumors, thus preventing progression and asphyxiation risk. The challenge in diagnosing thyroid nodules in the elderly is to screen for high-risk thyroid cancers, which is important for improving their prognosis. Cytopathology or even coarse needle biopsy is often insufficient (as in this case), and molecular testing may provide more assistance. To our knowledge, this is the first case of OCA with TERT promoter mutation as the initial presentation of asphyxia. This case aims to share the experience and contribute insights regarding the diagnosis and treatment of thyroid nodules in the elderly population.

In conclusion, this case highlights the successful management of a rapidly progressing thyroid tumor in an elderly patient. Swift surgical intervention, tailored to the patient’s age and comorbidities, led to a positive outcome. Molecular testing played a crucial role in identifying TERT promoter mutations. Despite postoperative hoarseness, the patient’s respiratory distress was resolved, underscoring the importance of individualized treatment for challenging thyroid nodules in elderly individuals.

## Data Availability

The original contributions presented in the study are included in the article/**Supplementary Material**. Further inquiries can be directed to the corresponding authors.
