# Supplementary figures and images for: Oncocytic cell carcinoma of the thyroid with TERT promoter mutation presenting as asphyxia in an elderly: a case report
Source: Front Endocrinol (Lausanne). 2024 Aug 16;15:1349114. doi: 10.3389/fendo.2024.1349114 (PMC11362092; doi:10.3389/fendo.2024.1349114)

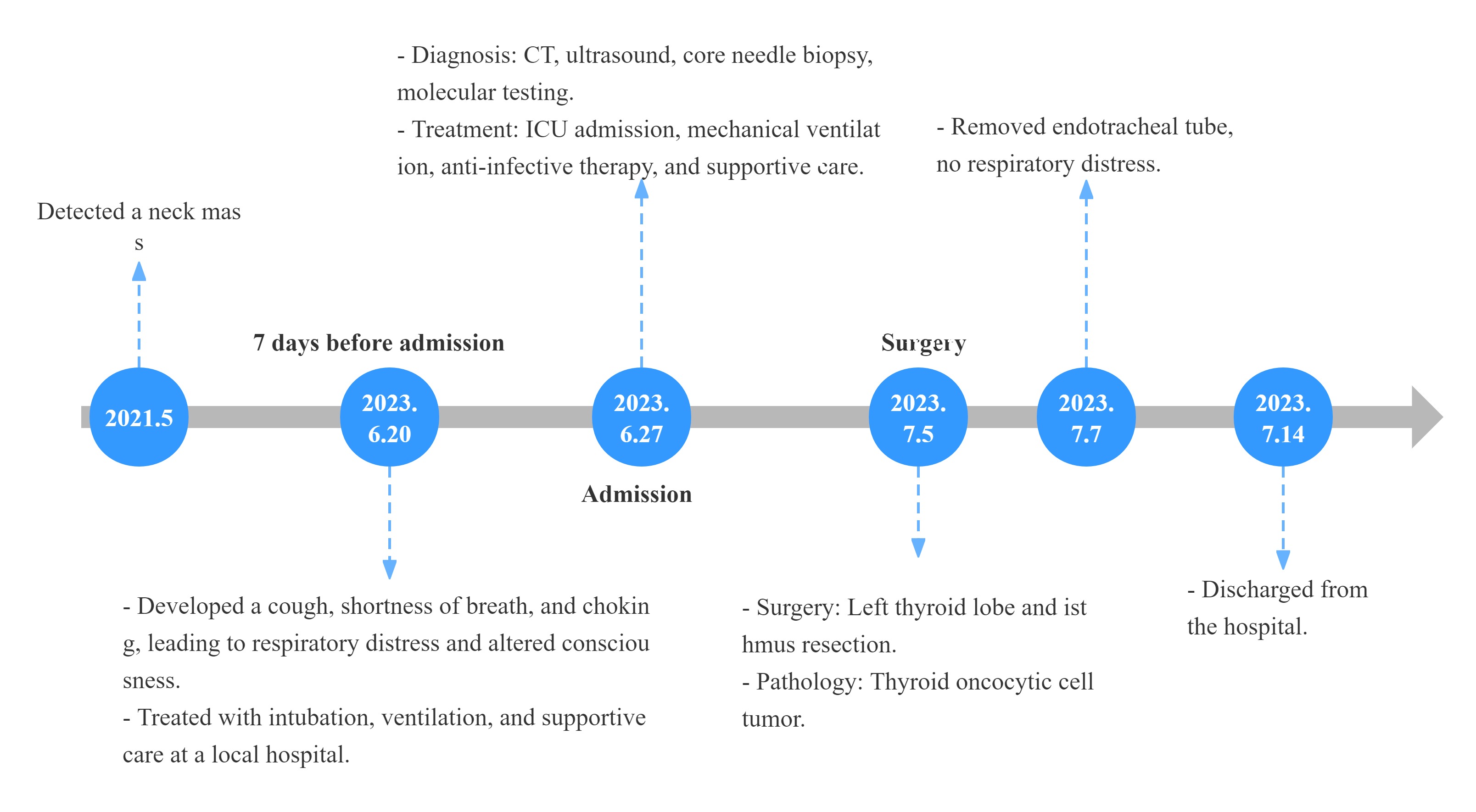

Supplement: Supplementary file 1 [file Image1.jpeg]
